# Technology-Based Interventions for Nursing Home Residents: Implications for Nursing Home Practice Amid and Beyond the Influence of COVID-19: A Systematic Review Protocol

**DOI:** 10.21203/rs.3.rs-56102/v2

**Published:** 2020-12-14

**Authors:** Zhaohui Su, Kylie Meyer, Yue Li, Dean McDonnell, Nitha Mathew Joseph, Xiaoshan Li, Yan Du, Shailesh Advani, Ali Cheshmehzangi, Junaid Ahmad, Claudimar Pereira da Veiga, Roger Yat-Nork Chung, Jing Wang

**Affiliations:** University of Texas Health Science Center at San Antonio; University of Texas Health Science Center at San Antonio; University of Rochester Medical Center; Institute of Technology Carlow; University of Texas Health Science Center at Houston; Beijing Normal University-Hong Kong Baptist University United International College; University of Texas Health Science Center at San Antonio; National Institute of Health; University of Nottingham; Peshawar Medical College; Universidade Federal do Parana; University of Hong Kong; University of Texas Health Science Center at San Antonio

**Keywords:** COVID-19, novel coronavirus, SARS-CoV2, nursing home residents, older adults, underserved populations, technology-based interventions, socio-ecological model

## Abstract

**Background:**

A growing number of technology-based interventions are used to support the health and quality of life of nursing home residents. The onset of COVID-19 and recommended social distancing policies that followed led to an increased interest in technology-based solutions to provide healthcare and promote health. Yet, there are no comprehensive resources on technology-based healthcare solutions that describe their efficacy for nursing home residents. This systematic review will identify technology-based interventions designed for nursing home residents and describe the characteristics and effects of these interventions concerning the distinctive traits of nursing home residents and nursing facilities. Additionally, this paper will present practical insights into the varying intervention approaches that can assist in the delivery of broad digital health solutions for nursing home residents amid and beyond the impact of COVID-19.

**Methods:**

Databases including PubMed, PsycINFO, CINAHL, and Scopus will be used to identify articles related to technology-based interventions for nursing home residents published between January 1^st^, 2010 to December 4^th^, 2020. Titles, abstracts, and full-texts papers will be reviewed against the eligibility criteria. The Preferred Reporting Items for Systematic Reviews and Meta-Analyses procedures will be followed for the reporting process, and implications for existing interventions and research evaluated by a multidisciplinary research team.

**Results:**

NA—protocol study

**Conclusions:**

Our study will fill critical gaps in the literature by providing a review of technology-based interventions tested in the nursing home setting. As the older adult population grows, there is an urgent need to identify effective technology-based interventions that can address the distinctive characteristics and preferences of nursing home residents. Clear and comprehensive understanding of how available technology-based health solutions facilitate healthcare for nursing home residents will shed light on the approaches open to residents to fend off the negative health consequences amid and beyond the influence of COVID-19.

**Systematic Review Registrations:**

PROSPERO CRD 42020191880

## Background

Nursing homes have been described as a "ground zero" throughout the coronavirus outbreak [1-4]. While the final impact of COVID-19 (coronavirus) on short and longer-term health outcomes is still unclear as the pandemic continues to unfold [5], what is clear is that nursing home residents have suffered some of the gravest consequences of this pandemic so far [6]. Contributing to over 40% of COVID-19 deaths within the United States, 100,033 residents and workers in nursing homes have passed away from COVID-19 (as of November 24^th^, 2020) [7]. Worse still is the fact that the nursing home residents who have died from COVID-19 mostly died without the care or company of their family members [8-10]. These staggering numbers underscore the urgent need for healthcare researchers to understand factors that make nursing home residents more vulnerable to COVID-19, and to identify practical solutions that can address these factors in a timely fashion.

Nursing homes and nursing facilities provide long-term service and support for individuals living with chronic or disabling conditions who are unable to live at home independently [11-13]. Often living with multiple morbidities, in the United States approximately 85.1% of nursing home residents are 65 years and older, with 75.8% of these individuals experiencing hypertension, 58.9% living with Alzheimer's Disease, and 53.0% of residents living with depression [13]. Three sets of factors likely contribute to the alarming COVID-19 death rates seen in nursing homes: (1) characteristics of nursing home residents [14-18], (2) distinctive attributes of nursing home facilities [1, 6, 9, 10, 19], and (3) the micro and macro-level supports available to nursing home residents [2, 20-24]. On a micro level, research indicates that nursing home residents are more susceptible to infection and fatal outcomes from COVID-19 because they are often older adults living with medical conditions that compromise the immune systems and lowers their ability to combat the virus [13, 19, 25, 26]. Additionally, they often lack specific self-care skills, such as utilizing telemedicine tools, or they may have a physical or cognitive impairment that impedes their ability to take care of their health and wellbeing [27-31]. The macro perspective focuses on the unique characteristics of nursing home facilities, as they are typically operating on a close and shared-living environment - conditions that are ideal for the spread of the virus [10, 19, 32]. Further, nursing homes often lack adequate healthcare resources or infrastructure needed to curb the impact of COVID-19. For example, numerous studies have indicated a lack of investment in training programs for nursing home staff, in addition to high turnover rates [33, 34], that management teams are often ineffective [35, 36], and that the nursing home infrastructure is often too outdated [32, 37-39].

The third set of factors that contribute to nursing home residents' vulnerability to COVID-19 centers on social support available to these adults. Nursing home residents often have limited access to micro-level social support, including support from formal (e.g., doctors and nurses) or informal caregivers (e.g., family, friends, and acquaintances) [40], local community [41], and organizations (e.g., inexperienced or inadequately trained staff) [42]. Furthermore, nursing home residents often have limited macro-level social support. This is evidenced by harmful social norms (e.g., age-related discrimination)[43-45] and inadequate policy support that facilitates healthy aging and quality of life (e.g., insufficient regulatory oversight to ensure quality care in nursing homes) [4, 20, 46, 47]. These factors combined, could result in severe health consequences in nursing home residents, such as wide viral spread [1].

**Figure 1:**
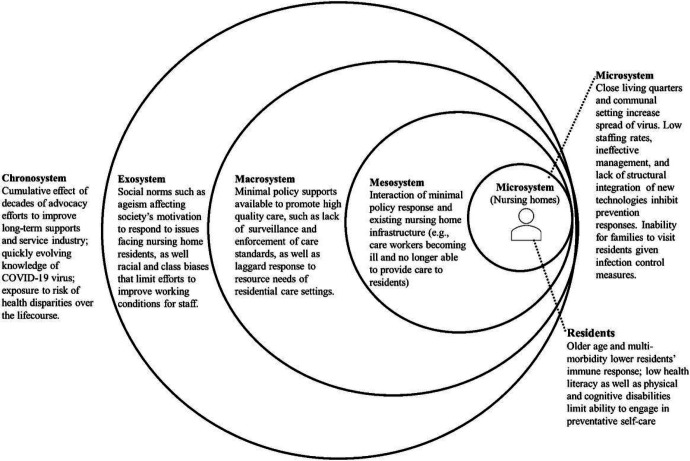
An ecological model of factors increasing nursing home resident vulnerability to COVID-19

The above areas of inquiry resonate with the core principles of the bioecological model [48-51], which highlights the way individuals are influenced by a series of synergistic interactions between intrapersonal and interpersonal factors (e.g., residents, resident families), organizational characteristics (e.g., nursing homes), policy (e.g., legislative response) and the social/community (e.g., ageism) context, and how these processes can change over time (See Figure 1.) To successfully and effectively protect nursing home residents from global health crises like COVID-19, stakeholders such as policymakers, healthcare professionals, informal caregivers, and older adults themselves all need to contribute to the change-making process [2, 46, 52, 53]. While some effective changes are resource-intensive, time-consuming, and need concerted efforts from multilevel stakeholders to achieve, there are cost-effective, efficient, and accessible health solutions available to nursing home residents, such as technology-based interventions [54, 55].

Technology-based interventions can be considered as the use of technology (e.g., digital devices like tablets and wearable devices, communication platforms) to manage or support health promotion strategies that aim to produce accessible and affordable health solutions to a target audience. Compared to traditional health solutions, such as face-to-face consultations, technology-based interventions have the potential to deliver healthcare more effectively and can mitigate geographic and access-related limitations that, as studies show, can play a significant role within nursing homes [56-62]. The evidence further suggests that technology-based interventions can help free healthcare professionals from repetitive work and allow them to make more meaningful contributions in delivering healthcare solutions to the care recipients [63-65].

Telemedicine and other technology-based solutions are particularly crucial given circumstances rendered by the COVID-19 pandemic, such as the limited ability for some healthcare providers to enter residences or for residents to visit their healthcare team for primary care visits. Limiting the exposure to infection through the use of telemedicine may assist in situations where a resident is required to attend a hospital appointment and return to a residence, thus alleviating the potential risk for a virus to spread to others [66]. Further, technologies that support residents' ability to remain in contact with families and friends outside of skilled care settings may reduce the adverse effects of loneliness and social isolation that are more common among nursing home residents compared to community-dwelling older adults [17, 67].

While technology-based solutions have potential to deliver health solutions to nursing home residents [68, 69], there is limited awareness of the benefits and delivery options for state-of-art technology-based interventions specifically designed for nursing home residents. By factoring for the distinctive characteristics of nursing home residents and nursing home facilities, the main focus of this systematic review is to identify and evaluate technology-based interventions tailored explicitly for nursing home residents. Additionally, this research will present practical insights into the varying intervention approaches that can assist in the delivery of broad digital health solutions for nursing home residents amid and beyond the impact of COVID-19. Overall, our research aims are:

- To identify technology-based interventions designed for nursing home residents and describe the characteristics and effects of these interventions concerning the distinctive traits of nursing home residents and nursing facilities.
- To present practical insights into the varying intervention approaches that can assist in the delivery of broad digital health solutions for nursing home residents amid and beyond the impact of COVID-19.

## Methods

The Preferred Reporting Items for Systematic Reviews and Meta-Analyses (PRISMA) procedures will be adhered to in the reporting process [70]. This systematic review is registered with the International Prospective Register of Systematic Reviews (PROSPERO) system (CRD 42020191880); these measures are to avoid unnecessary study duplication [71, 72], increase research rigor [73, 74], improve study comparability and replicability [75], and ultimately, promote quality and transparency in research [76]. The review being carried out in line with the Cochrane Handbook [77].

### Inclusion and exclusion criteria

Based on the research aims, inclusion criteria were set a priori (Table 1). Articles published between January 1^st^, 2010 to December 4^th^, 2020 were included in the review. This period of time was selected to ensure a focus on up-to-date technologies, given the tendency of technology-based interventions to evolve and become out-of-date. The timeline will also make sure both studies conducted before and amid COVID-19 will be included in the review. In this study, nursing home residents are defined as people “having a length of stay in a nursing home for more than 90 days at any point” [78]. Technology-based interventions are defined as “the use of technology to manage or support health promotion strategies that aim to produce accessible and affordable health solutions to a target audience” [79]. Articles will be excluded if (1) the study sample did not include a majority nursing home residents (i.e., nursing home residents ≤ 50% of the total research population), (2) the study did not include and discuss technology-based health solutions designed for nursing home residents, (3) the study did not adopt a randomized controlled trial design (e.g., studies with quasi-experimental design were excluded), and (4) the authors did not report original empirical findings on intervention outcomes (e.g., research protocols and systematic review studies were excluded from the review).

### Search strategy

Databases including PubMed, PsycINFO, CINAHL, and Scopus, will be searched for eligible articles. We will also search ProQuest Dissertations to examine gray literature sources. A search strategy was developed in consultation with a librarian experienced in systematic review methods. Search terms used to locate articles will center on three concepts: nursing home residents, technology-based interventions, and randomized controlled trials. An example PubMed search string is illustrated in Table 2.

### Study selection

Following the search, all citations will be collated and uploaded to Mendeley, and duplicate studies will be removed. Titles and then abstracts will be screened by two principal reviewers independently. The same screening procedure will be adopted in the full-text article review process on selected article abstracts. Reasons for exclusion will be recorded and detailed in the PRISMA flowchart. Discrepancies between reviewers will be resolved via group discussions using videoconferencing and email correspondence to reach a consensus.

### Study quality assessment

The Cochrane Collaboration evaluation framework will be adopted to examine risk of bias of the included study [80]. The framework has seven domains: random sequence generation, allocation concealment, blinding of participants and personnel, blinding of outcome assessment, incomplete outcome data, selective reporting, and any other source of bias. The risk of bias will be evaluated independently by two reviewers (ZS and XL), who will qualitatively evaluate risk of bias and provide a score (high, medium, low). Any discrepancy regarding the risk of bias will be resolved by consensus via group discussions. Both junior and senior authors will be involved in the discrepancy review process, wherein, if agreement cannot be reached, the senior author will decide on the appropriate rating.

### Data extraction and synthesis

Data on study design, sample characteristics (i.e., sample size and sample details), intervention characteristics (i.e., technology type, intervention application, intervention exposure, and intervention materials), outcome variables assessed, and research findings will be extracted by two main reviewers (ZS and XL). Findings from the included studies will be narratively synthesized to examine the characteristics and effects of the interventions. Gaining a more structured understanding of the interventions, the multidisciplinary study team will organize insights on intervention application and outcomes in tandem with the distinctive traits of nursing home residents and the overall nursing home environment. Due to the heterogeneity found within the articles identified during a preliminary review of search results, meta-analyses are not considered.

## Results

NA: As this is a protocol study

## Discussion

There is a growing body of technology-based interventions designed to support the health and quality of life of nursing home residents [56-62]. The onset of COVID-19 and recommended social distancing policy led to an increased interest in reliance on technology-based solutions [81, 82]. However, research has yet to provide comparative insight into the recent state of development of these interventions and how current evidence apply in the context of the COVID-19 pandemic. The use of the socio-ecological model, combined with multidisciplinary expertise, provides a framework to present practical insights on how these interventions can be utilized to deliver health solutions to nursing home residents amid and beyond the impact of COVID-19.

This research fills a critical gap in the literature by consolidating, in one place, the evidence for technology-based interventions empirically tested with nursing home populations. As the older adult population grows, there is an urgent need to identify effective technology-based interventions that can address the distinctive characteristics and preferences of nursing home residents [83, 84]. Improving person-centered care and the delivery of effective care solutions to nursing home residents, especially as the pandemic continues, is of critical importance. Comprehensive understanding of how available technology-based health solutions facilitate healthcare for nursing home residents can help shed light on approaches that are available to these residents to fend off the negative health consequences amid and beyond the influence of COVID-19. While the COVID-19 pandemic has revealed troubling vulnerabilities in the long-term care system across the globe, it also shows how telemedicine can support nursing home residents and their families. Technology can also assist clinicians in connecting with patients when in-person medical visits are difficult or dangerous (e.g., in rural settings, following natural disasters). Telemedicine and other technology-based interventions have the potential to provide a comprehensive range of benefits. This research also serves as a platform for care institutions and policymakers to inform government policies and further justify the role technology can play in strengthening the service provision across nursing homes and facilities.

## Conclusions

NA: As this is a protocol study

## Figures and Tables

**Table 1.** Study inclusion criteria

Category	Criteria
Study population	Adults (≥18 years old) living in nursing homes
Language	English and Chinese
Intervention/health solution	Technology-based interventions (e.g., digital tools such as smartphones and tablets, sensor devices, internet-based programs)
Key variable	Detailed descriptions of the technology-based interventions (i.e., purpose of the intervention (to evaluate the aims of existing interventions), use of technology (to examine how different types of technologies have been applied among nursing home residents), application of the interventions (to investigate the degree to which the interventions have involved nursing home residents in the adoption/application of technologies), intervention exposure (to study how different interventions have been used among nursing home residents), outcome variables assessed/measured (to evaluate the quantifiable outcomes of technology-based interventions), and weather the design of the intervention material is tailored to nursing home residents (to examine to degree to which the design technology-based interventions has taken the unique attributes of nursing home residents into consideration)
Study type	Original research (i.e., research that reports original and empirical research findings)
Study design	Randomized controlled trials
Study outcome	Empirical reporting of the effect of the intervention (i.e., qualitative designs excluded)

**Table 2.** Example PubMed search string

Concept	Search string
Nursing homes	“nursing home”[MeSH] OR “nursing home”[TIAB] OR “nursing homes” [MeSH] OR “nursing homes” [TIAB] OR “residential home*” [MeSH] OR “residential home” [TIAB] OR “caring home*” [TIAB] OR “home for the aged” [MeSH] “home for the aged” [TIAB] OR “long term care” [MeSH] OR “long term care” [TIAB] OR “senior housing” [TIAB] OR “assisted living facilities” [MeSH] OR “assisted living facilities”[TIAB] OR (senior[TIAB] OR geriatric[TIAB] OR elderly[TIAB] OR aged[TIAB] OR elder[TIAB] “older adults” [TIAB]) AND (housing[TIAB] OR living[TIAB])
Technology-based Interventions	“technology” [MeSH] OR “technology” [TIAB] OR “eHealth”[TIAB] OR “telemedicine” [MeSH] OR “telemedicine” [TIAB] OR “tele-medicine” [MeSH] OR “tele-medicine” [TIAB] OR “telehealth” [TIAB] OR “tele-health” [TIAB] OR “connected health” [TIAB] OR “digital health” [TIAB] OR “mHealth”[TIAB] OR “mobile health” [TIAB]
Randomized controlled trials	randomized controlled trial[PT] OR randomized controlled trials as topic[MH] OR random allocation [MH] OR double-blind method[MH] OR single-blind method[MH] OR random*[tw] OR "Placebos"[MeSH] OR placebo[TIAB] OR ((singl*[tw] OR doubl*[tw] OR trebl*[TW] OR tripl*[TW]) AND (mask*[TW] OR blind*[TW] OR dumm*[TW]))

## List Of Abbreviations

COVID-19: covronavirus disease 2019
